# Elimination Diets in Lactating Mothers of Infants with Food Allergy

**DOI:** 10.3390/nu16142317

**Published:** 2024-07-18

**Authors:** Mariannita Gelsomino, Lucia Liotti, Simona Barni, Francesca Mori, Mattia Giovannini, Carla Mastrorilli, Luca Pecoraro, Francesca Saretta, Riccardo Castagnoli, Stefania Arasi, Angela Klain, Michele Miraglia del Giudice, Elio Novembre

**Affiliations:** 1 Pediatric Allergy Unit, Department of Life Sciences and Public Health, University Foundation Policlinico Gemelli IRCCS, Catholic University of the Sacred Heart, 00168 Rome, Italy; 2 Pediatric Unit, Department of Mother and Child Health, Salesi Children’s Hospital, 60123 Ancona, Italy; lucialiotti@libero.it; 3 Allergy Unit, Meyer Children’s Hospital IRCCS, 50139 Florence, Italy; simonabarni@hotmail.com (S.B.); francesca.mori@unifi.it (F.M.); mattiag88@hotmail.it (M.G.); 4 Department of Health Sciences, University of Florence, 50139 Florence, Italy; elio.novembre@unifi.it; 5 Pediatric Hospital Giovanni XXIII, Pediatric and Emergency Department, AOU Policlinic of Bari, 70126 Bari, Italy; carla.mastrorilli@gmail.com; 6 Pediatric Unit, Department of Surgical Sciences, Dentistry, Gynecology and Pediatrics, University of Verona, 37126 Verona, Italy; luca.pecoraro@aovr.veneto.it; 7 Pediatric Department, Latisana-Palmanova Hospital, Azienda Sanitaria Universitaria Friuli Centrale, 33100 Udine, Italy; francescasaretta@gmail.com; 8 Department of Clinical, Surgical, Diagnostic and Pediatric Sciences, University of Pavia, 27100 Pavia, Italy; riccardo.castagnoli@yahoo.it; 9 Pediatric Clinic, Fondazione IRCCS Policlinico San Matteo, 27100 Pavia, Italy; 10 Translational Research in Pediatric Specialties Area, Division of Allergy, Bambino Gesù Children’s Hospital, IRCCS, 00165 Rome, Italy; stefania.arasi@opbg.net; 11 Department of Woman, Child and General and Specialized Surgery, University of Campania “Luigi Vanvitelli”, 80138 Naples, Italy; klainangela95@gmail.com (A.K.); michele.miragliadelgiudice@unicampania.it (M.M.d.G.)

**Keywords:** food allergy, lactating mothers, exclusively breastfed infants

## Abstract

Breastfeeding is the most important nutrition source for infants. However, managing breastfed infants with signs and symptoms related to food allergy can be difficult. Many studies have shown the presence of different food allergens in breast milk, but the clinical role of these antigens in human milk is still much debated. Milk is the main suspect in exclusively breastfed infants with signs and symptoms attributable to food allergy, even if other foods may be responsible. This narrative review analyzes the recommendations provided by international guidelines to determine the diagnosis and management of IgE-mediated and non-IgE-mediated food allergies in exclusively breastfed infants. Dietary restrictions in lactating mothers of infants with suspected FA are usually not necessary. Only in the very few cases where significant allergy signs and symptoms occur in the infant during exclusive breastfeeding should the lactating mother follow an elimination diet for the suspected food for a short period.

## 1. Introduction

Breastfeeding is the most important nutrition source for infants. Still, managing breastfed infants with signs and symptoms related to food allergy (FA) can be difficult. It is essential to assess whether the clinical manifestations infants present are due to FA and whether they could benefit from an elimination diet prescribed to their mothers. There are many foods that could be the cause of the onset of symptoms that could be an expression of both IgE- and non-IgE-mediated forms of allergy, so skin prick tests and serum-specific IgE assays may not always be helpful in diagnosis. Many studies in the literature have investigated the passage of allergens through breast milk and the molecular mechanism behind this transfer, although their action on modulating the immune system of exclusively breastfed infants is not yet known.

This narrative review intends to offer an overview of the data concerning the transfer of food allergens in human milk and their possible role in food allergies. Furthermore, this manuscript analyzes the recommendations provided by international guidelines (summarized in Table 1) to establish the diagnosis and management of IgE-mediated and non-IgE-mediated FA in exclusively breastfed infants. 

## 2. Transfer of Food Allergens into Human Milk

**Figure 1:**
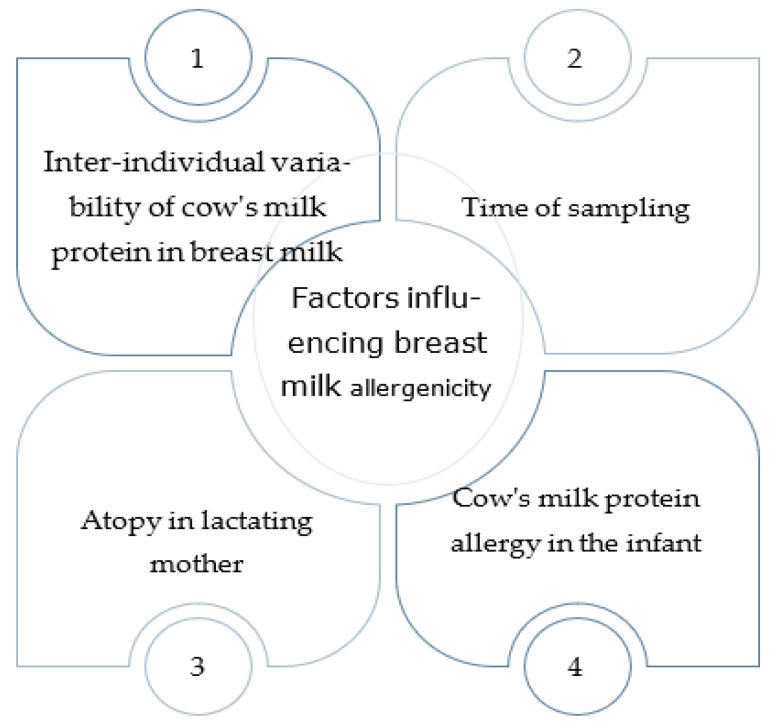
Factors influencing the transfer of cow’s milk proteins into breast milk and allergy to breast milk.

Many studies have demonstrated the existence of several food allergens in breast milk, including betalactoglobuline [8,9,10,11,12,13], casein [14], ovalbumin [12,15], ovomucoid [16], gliadin [17,18], and peanut proteins [19,20,21]. The clinical role of these antigens in human milk is still much debated because the measured concentrations are often extremely low. Also, it is still not known whether allergens transferred to the infant through breastfeeding play a protective role in atopy or are responsible for sensitization. Before they get to the mammary gland, food antigens transferred from the mother’s diet have passed through the mother’s gut, where digestive enzymes could have generated antigen variants with tolerogenic properties [22]. Moreover, human milk predigestion within the mammary gland may be critical for generating tolerogenic (or immunogenic) peptides [23]. However, the first exposure to a food allergen might be crucial for the breastfed baby’s sensitization or development of oral tolerance. The amounts measured vary greatly depending on the allergen, inter-individual variability, and timing of the sample collection after exposure. Regarding proteins included in cow’s milk, 15 to 47% of women have no detectable beta-lactoglobulin values 1, 3 after consumption, and the peak concentration is highly variable, but there is no association between the amount ingested and the amount detected [9,10,11]. Sorva et al. [10] reported a marked variation in the measurable concentration of beta-lactoglobulin in breast milk, even though all women were sampled 1–2 h after ingesting the same amount of cow’s milk (400 mL). A Swedish study [11] also showed no association between cow’s milk consumption and the amount of beta-lactoglobulin secreted in breast milk, with concentrations varying widely from 5 to 800 ng/mL. In contrast, Fukushima et al. [12] suggest that there might be a correlation between beta-lactoglobulin concentrations in human milk and long-term consumption of cow’s milk. In fact, lower beta-lactoglobulin concentrations were found in the milk of women who had consumed low amounts of cow’s milk and dairy throughout the lactation period, even if all participants had consumed the same daily amount of cow’s milk (200 mL) for one week prior to sampling. Another factor that could influence the secretion of beta-lactoglobulin by the mammary glands could be the mother’s allergic status, as suggested by Dekker et al. [24]. Their study showed a higher concentration of beta-lactoglobulin in allergic mothers’ breast milk, probably due to the higher intestinal absorption. Furthermore, another study [10] reports that the secretion pattern of beta-lactoglobulin may be very different between mothers of infants presenting cow milk protein allergy (CMPA) and mothers of healthy ones depending on different intestinal macromolecule absorption. However, the variability in beta-lactoglobulin concentrations may also be partly due to the different measurement methods used, as the sensitivity of methods that detect peptides from gastric digestion is greater than that of methods detecting the whole proteins [25]. In addition, regarding the concentration of beta-lactoglobulin, the detection time is also greatly variable; it can be detected in breast milk from 1 to 2 h after ingestion of cow’s milk to 10 days after the cow’s milk protein is removed from the mother’s diet [26]. Therefore, multiple factors influence the transfer of cow’s milk proteins into breast milk (Figure 1).

Nonetheless, according to some studies, the concentration of milk/lactoglobulin in breast milk ranges from 0.01 to 800 ng/mL, and, theoretically, in a few cases, in a single meal, it could reach sufficient amounts to trigger an allergic reaction in the infant [27]. Regarding chicken egg proteins, it has been reported that approximately 25 per cent of women who consume eggs have no detectable values of ovalbumin in their milk [28,29]. Despite this, a blinded trial performed by Palmer et al. [29] shows that there may be a direct correlation between the concentration of ovalbumin in breast milk and the amount of ovalbumin ingested by the mother. Also, the type of egg processing could influence the transfer of the proteins into breast milk. A higher concentration of ovalbumin is reported in breast milk after ingesting cooked egg rather than raw egg. The study by Hirose et al. [16] also reports that ovalbumin (molecular weight 28 kDa), detected in 12 out of 37 samples, occurs in breast milk with a much higher molecular weight (450 kDa), suggesting that this protein is present as an immune complex with IgA. Gliadin can also be detected in human milk as immune complexes (gliadin/IgA-antigliadin) or non-degraded gliadin [17]. Its concentrations are very high compared to the other food antigens in human milk, but the amounts detected are highly variable. According to data reported by Chirdo et al. [18], the concentration of gliadin varies between 5 and 1200 ng/mL, and significantly lower values are observed in serum (mean 41 ng/mL) than in colostrum and milk (mean 883 ng/mL). However, there seems to be no correlation between serum, milk, and colostrum concentration within the same individual, nor between its levels and amount of gluten intake. Furthermore, in the study, gliadin was also found in the milk of six women who had followed a gluten-free diet for 3 days, suggesting that a short-term elimination diet does not lead to the disappearance of the allergen from the mother’s milk. Furthermore, Troncone et al. [17] demonstrated that the gliadin concentration in breast milk varies depending on the time elapsed since the ingestion of gluten; the peak is observed at 2–4 h. Finally, regarding peanut allergens, several studies have demonstrated the transfer of Ara h 2 and Ara h 6 into breast milk. Ara h 2 has a range of 2.3–184 ng/mL, while the Ara h 6 level varies from 1.1 to 79 ng/mL in the milk of women on a free diet. The two proteins become detectable in human milk simultaneously but at different concentrations (Ara h 2 is present at higher concentrations than Ara h 6). After ingestion of peanuts, 52% of the women had no detectable peanut protein levels in milk [19], and in 72% [20] of them, no Ara h 2 was found. Considering all of these studies, the nutritional composition of breast milk appears to be dynamic and to depend on several factors, including not only the maternal diet but also extreme inter- and intra-individual variability in the transfer of food allergens into human milk. The number of allergenic foods in human milk is very low, and their amount is rarely capable of inducing an allergic reaction. In fact, 5% of the allergic population is likely to respond with objective reactions at the protein dose of 1.6 mg for peanut, 7.4 mg for cashew nut, 1.1 mg for cow’s milk, 1.5 mg for hen’s egg, and 0.29 mg for hazelnut [30].

## 3. Management of Suspected IgE-Mediated FAs in Exclusively Breastfed Infants

Milk is the main suspect in exclusively breastfed infants with signs and symptoms attributable to FA, even if other foods may be responsible. The most common FA in infancy is CMPA, for which the prevalence ranges from 2% to 7.5% [31,32] in the first year of life. The most important strategies to manage CMPA recommended by international guidelines [1,3,6,7,33] are to continue breastfeeding as the best nutrition source for infants with allergies, as they commonly tolerate the breast milk of mothers on a free diet. Munblit et al. [34] estimated that, for over 99% of infants who are diagnosed CMPA, mothers consuming cow’s milk produce breast milk with not enough allergen to trigger an allergic reaction. McWilliam et al. [6] suggest that maternal elimination trials can be considered in IgE-mediated FA only when the infants are symptomatic on breastfeeding alone. In general, if IgE-mediated CMPA is suspected in breastfed-only infants, according to the 2012 ESPGHAN guidelines [32], a cow’s-milk-protein-elimination diet should be indicated to lactating mothers for a period ranging from 3 to 5 days [35] to obtain diagnostic confirmation.

Regarding other IgE-mediated FAs, reports of adverse reactions following the ingestion of human milk are rare. For example, cases of immediate reactions (within one hour after breastfeeding) occurring several hours after the mother ingested fish with subsequent positive prick tests and/or specific IgE for fish in the infant have been reported in the literature [36,37]. No additional adverse reactions occurred after the mother started a fish-free diet in these cases. In both of these reported cases, the challenge was not carried out. In the first case [36], however, the reappearance of urticaria was described in the infant after breastfeeding upon the mother’s accidental ingestion of fish about one month after the diagnosis. In the second case [37], the diagnosis was also made solely based on the clinical history (mother’s ingestion of trout after which the anaphylactic reaction had occurred) and the detection of sensitization in the infant. Moreover, the child subsequently presented systemic reactions during weaning within minutes of ingesting small amounts of salmon and anchovy. Although other reports [8,38,39,40,41] published in the literature suggest that food proteins, such as cow’s milk, chicken’s egg, wheat, and peanut, present in human milk may trigger IgE-mediated allergic reactions during lactation according to the 2022 systematic review by Gamirova et al. [42], there is an estimated ≤1:1000 probability of an IgE-mediated allergic reaction to these allergens in infants during breastfeeding. In fact, a comparison was carried out between the data regarding the food protein level detected in human milk and those taken from the Voluntary Incidental Trace Allergen Labelling (VITAL 3.0) guide. The aim was to assess the probability of food-allergic individuals experiencing an immediate-type allergic reaction upon breast milk ingestion. All food protein levels across the studies were considerably lower than the eliciting dose of 1% for allergic individuals (ED01) in most of the samples.

## 4. Management of Suspected Non-IgE-Mediated FAs in Exclusively Breastfed Infants

### 4.1. Gastrointestinal Clinical Manifestations and Eczema in Exclusively Breastfed Infants

Even for non-IgE-mediated FAs, the main suspected food is cow’s milk, and only a little evidence is available in the literature regarding their occurrence in exclusively breastfed infants. In fact, in the European Association of Allergy and Immunology (EAACI) Position Paper of 2019 [4], it is stated that breastfeeding is also recommended for infants with suspected non-IgE-mediated FAs. Nevertheless, there are some studies [43,44] that have reported the occurrence of non-IgE-mediated CMPA during exclusive breastfeeding (among infants who have never consumed cow’s milk protein), with a prevalence of 0.4–0.5%. In the prospective study by Jakobsson et al. [43] conducted in the Swedish population, it was reported that out of the 20 children diagnosed with CMPA in their first year of life, 4 presented signs and symptoms (colic, vomiting, diarrhea, rash, and eczema) during exclusive breastfeeding; the clinical manifestations disappeared with a cow’s-milk-protein-elimination diet in the lactating mother and reappeared after the reintroduction of cow’s milk protein in the maternal diet. The same signs and symptoms recurred after directly administering cow’s milk protein to these children. The study did not carefully investigate the possible exposure to cow’s milk protein in the very early neonatal period. In contrast, Host et al. [44] reported that all nine infants with non-IgE-mediated CMPA who were exclusively breastfed had been exposed to very small amounts of milk formula (0.3–4 g of beta-lactoglobulin) in the first 3 days of life. This could indicate that early and sporadic exposure to cow’s milk protein could trigger sensitization in predisposed infants, who would then develop clinical manifestations after consuming small amounts of cow’s milk protein in breast milk. Again, the signs and symptoms resolved by removing cow’s milk and dairy products from the mother’s diet for 4 weeks and reappeared after the challenge (reintroduction of at least 0.5 L of cow’s milk in the mother’s daily diet), mainly as gastrointestinal and cutaneous clinical manifestations. Thus, the most frequent clinical manifestations of non-IgE-mediated CMPA in breastfed infants are mainly gastrointestinal, such as regurgitation, colic, and irritability, and skin clinical manifestation, such as atopic dermatitis [31], whereas severe reactions, such as severe atopic eczema or protein-losing enteropathy, are extremely rare [45]. In general, if non-IgE-mediated CMPA is suspected in exclusively breastfed infants because of certain delayed signs and symptoms, according to the 2012 ESPGHAN guidelines [32], a cow’s-milk-protein-elimination diet should be prescribed to lactating mothers for a period of 2–4 weeks [35,46] to obtain diagnostic confirmation. If clinical manifestations disappear after this period, cow’s milk should be included again in the maternal diet (challenge with small, medium, or large amounts of allergen according to the clinical reactivity of the infant). If no improvement in signs and symptoms is observed during the elimination diet period or if the child remains without clinical manifestations despite the challenge, then the breastfeeding mother should return to a free diet because the diagnosis of CMPA would be ruled out. If, on the other hand, signs and symptoms recur, the diagnosis of CMPA is confirmed, and then cow’s milk protein should be excluded from the lactating mother’s diet. In the case of a prolonged elimination diet for the mother, supplementation with calcium and vitamin D is recommended, while supplementation with iodine and vitamin B12 can be taken into consideration [7]. Moreover, in the case of a prolonged maternal elimination diet, periodic attempts at allergen reintroduction should be considered for all lengths of breastfeeding. If CMPA is confirmed and breast milk becomes insufficient or the mother can no longer breastfeed for other reasons, the first choice of formula to be used instead of breast milk is extensively hydrolyzed formula (EHF), as at least 90% of children with CMPA tolerate it [47,48].

Conversely, amino acid formulas (AAFs) should be indicated only if signs and symptoms persist for 2–4 weeks after starting the EHF [46]. Persistence of clinical manifestations despite using an AAF rules out the diagnosis of CMPA.

The same management exemplified above may be applied for all suspected culprit foods causing gastrointestinal signs and symptoms or eczema.

### 4.2. Food-Protein-Induced Allergic Proctocolitis in Exclusively Breastfed Infants

Food-protein-induced allergic proctocolitis (FPIAP) is an immune-mediated inflammation of the distal colon caused by exposure to one or more food antigens; the signs and symptoms tend to appear during the first months of life. In FPIAP, recto sigmoidoscopy could show mucosal congestion with petechial areas [49]. The inflammation is characterized by eosinophilic infiltration and lymphonodular hyperplasia [50]. Its prevalence can vary widely, from 0.16% in apparently healthy infants to 64% in infants with rectal bleeding [51,52]; this is because many infants with rectal bleeding diagnosed with FPIAP actually may not have FPIAP and therefore go through unnecessary changes in maternal diet or the use of expensive and unnecessary formulas, perhaps even discouraging breastfeeding [51]. Cow’s milk proteins are the main reason behind the development of FPIAP (65% of cases), but other foods may be implicated, such as chicken eggs (19% of cases), soya (6% of cases), and wheat (3% of cases) [5]. In addition, a multiple-food allergy has been identified in around 5% of infants [53]. Over 50% of FPIAP cases reported in the literature involve exclusively breastfed infants [5]. The clinical management of FPIAP in exclusively breastfed infants consists of eliminating the suspected culprit food from the mother’s diet. Rectal bleeding typically resolves macroscopically within 72–96 h from the start of rigorous elimination of the offending protein from the mother’s diet, with complete disappearance within 1–2 weeks. If the clinical manifestations persist despite the cow’s-milk-protein-free diet, monitoring the mother’s adherence to the diet becomes necessary; secondly, first soy and then chicken egg shall be removed from her diet [54]. As shown in the protocol for the diagnosis and management of FPIAP proposed by Mennini et al. [5], if the rectal bleeding has resolved, it is necessary to reintroduce the suspected food into the maternal diet to assess the reappearance of the bleeding within 72–96 h [55]; if the signs and symptoms reappear, the diagnosis is confirmed. It is therefore suggested that the mother continue the diet without the culprit food. The timing for the reintroduction of the offending food varies widely among the different studies. It usually takes place at one year of age, yet some authors [5,56] based on the study by Elizur et al. [53] suggest an earlier reintroduction. In fact, in one study [53] on 21 children (mean age at presentation was 52.9 ± 49.6 days) with hematochezia and CMPA, almost 80% of the children had cow’s milk reintroduced into their diet at an average age of 5.3 ± 2.1 months without any adverse events. Therefore, CM reintroduction after 3 months on the diet is recommended [5,56]. In the study by Martin et al. [57], tolerance is also established after an average of 50 days. Still, the diagnosis of FPIAP is overestimated, as it also considers occult blood alone as a sufficient sign when diagnosing FPIAP. Overall, a 3-month exclusion period seems acceptable, and longer reintroduction times could be considered for more severe cases or for the ones associated with IgE-mediated FA, where tolerance has been shown to set in later [58,59]. In a randomized clinical trial published in 2005 by Arvola et al. [52], the usefulness of the elimination diet in the case of rectal bleeding in infants was put in doubt. Forty children were enrolled, with 68% exclusively breastfed and allocated to two arms: nineteen children (or their mothers) followed the cow’s-milk-protein-elimination diet, while twenty-one continued their (or their mother’s) free diet. An elimination diet and subsequent challenge diagnosed CMPA in only two patients. On average, the cow’s-milk-protein-elimination diet did not change the duration or severity of rectal bleeding. In conclusion, the challenge is necessary for children who resolved their clinical manifestations during the elimination diet period to reduce the cases of misdiagnoses or unnecessary diets. Occasionally, a recurrence of signs and symptoms may be observed, probably due to the mother’s accidental ingestion of small amounts of the trigger food or other causes of rectal bleeding if FPIAP is not involved, the latter being not so uncommon according to some authors [5,51,60]. For infants who do not respond to the mother’s dietary restrictions, then, only if the culprit food cannot be identified, some authors [60] suggest an attempt to discontinue breastfeeding by switching to EHF, and some suggest switching to an AAF if symptoms persist despite EHF [61,62]. Discontinuation of breastfeeding is not supported by other authors [57], who propose an algorithm that advises if the rectal bleeding persists despite one week of a cow’s-milk-, soya-, and hen’s-eggs-elimination diet to resume a free diet for the breastfeeding mother and not to take any other additional measure, at least until the infant is one year old, considering an allergic cause is unlikely and taking into great consideration the child’s state of well-being (an essential condition for suspecting FPIAP). Indeed, with no warning signs and in otherwise well-appearing infants, it is possible to think of other possible causes of rectal bleeding, such as benign non-allergic infantile proctocolitis [63]. In general, in the case of the failure of the breastfeeding mother’s elimination diet, the opportunity to switch to EHF or the indication to resume a free diet tolerating hematochezia should be discussed with the parents [62]. If the bleeding persists beyond one year of age (an arbitrarily chosen time) or there are warning signs (e.g., anemia, failure to thrive, abdominal pain, severe perianal disease, and deteriorating general condition [5]), it would be advisable to proceed with other non-allergological exams [64], e.g., a colonoscopy with biopsy.

### 4.3. Food-Protein-Induced Enterocolitis Syndrome in Exclusively Breastfed Infants

Food-protein-induced enterocolitis syndrome (FPIES) is a non-IgE-mediated FA (incidence between 0.015% and 0.7% [65]) that can manifest in an acute form, consisting of repeated vomiting associated with pallor, lethargy, and sometimes diarrhea, with onset 1–4 h after ingestion of the trigger food [2]. It can also manifest in a less common, chronic form. Chronic FPIES usually occurs in infants fed with cow-milk-protein- or soy-based formulas, and it is clinically characterized by intermittent vomiting, diarrhea, lethargy, dehydration, and failure to thrive [66]. The main cause of FPIES is usually the direct ingestion of the offending food by the child, but the relevant literature also includes cases of chronic FPIES from cow’s milk protein [67,68] and a case of acute FPIES from soy [69] in exclusively breastfed infants due to the passage of the offending protein through the mother’s milk. In all patients, the signs and symptoms resolved after the culprit-food-elimination diet started. Although guidelines [70] suggest performing an oral food challenge (OFC) at least 2 weeks after commencing the elimination diet to confirm the diagnosis of chronic FPIES, in none of these cases reported in the literature the OFC was performed due to the severity of the episode according to the patient’s or parents’ wishes. In other reported cases of acute FPIES to cow’s milk in exclusively breastfed infants, no confirmatory OFC was performed [71]. In the described cases of FPIES to cow’s milk [68] and soya [69], it was also reported that there was no recurrence of clinical manifestations even though the lactating mother continued to consume bakery products containing cow’s milk and soya, respectively.

Concerning culprit food traces, guidelines [2] report that it is not necessary to routinely recommend avoiding products with precautionary allergen labels in patients with FPIES, and no cases of breast-milk-induced FPIES after ingestion by the lactating mother of products with a precautionary label for the culprit food have been reported in the literature. Only one case [67] of clinical manifestations’ recurrence in an exclusively breastfed infant due to the mother’s ingestion of very small amounts of cow’s milk protein (butter sauce) has been reported in the studies carried out so far.

Therefore, the probability of chronic FPIES in exclusively breastfed infants is extremely low and not well-demonstrated in the literature, and the breastfeeding mother’s diet appears to be implicated in the few cases with a consistent history of reactions triggered by breast milk [2]. Moreover, the breastfeeding mother’s diet should not be strict (without products with precautionary allergen labeling), according to the cases described by Monti [68] and Tan [69] in which the lactating mother continued to consume bakery products without recurrence of signs and symptoms.

### 4.4. Food Protein Enteropathy and Gastritis in Exclusively Breastfed Infants

Finally, regarding food protein enteropathy (FPE), a type of inflammation of the small intestine that appears in the first 2–9 months of life, typically within a few weeks of the introduction of the offending food (usually cow’s milk or soya) [54], characterized by protracted diarrhea, malabsorption, hypoalbuminemia, and failure to thrive, it does not generally occur in exclusively breastfed children with a mother on a free diet. However, in suspected cases, a trial elimination diet may be performed to assess the response of chronic gastrointestinal clinical manifestations to the mother’s dietary restrictions. Signs and symptoms generally resolve within 1–4 weeks [72], and, in such cases, reintroducing the allergen into the mother’s diet or performing an OFC is necessary to ensure correct diagnosis and the need to continue the exclusion diet [4]. Only four case reports [73,74,75,76] of suspected allergic enteropathies in exclusively breastfed infants are reported in the literature, and, of these, in only one [68] was FPE confirmed by biopsy.

Higuchi et al. [73] report a case of a protein-dispersive enteropathy with loose stools and poor weight gain in an exclusively breastfed infant. Specific IgE to yolk and albumen (overlap forms) were detected and led to eliminating chicken eggs from the lactating mother’s diet, with the resolution of the clinical manifestations and reappearance after eggs were introduced into the baby’s diet. The second case [76] involved an exclusively breastfed infant suffering from protein-dispersive enteropathy and severe atopic dermatitis. After the elimination of hen’s egg, cow’s milk, wheat, and peanuts from the mother’s diet (again following the detection of specific IgE to these foods), signs and symptoms resolved completely. In this case, a diagnostic challenge was not performed. A similar case was reported by Kondo et al. [74], with recovery of FPE after removing eggs from the maternal diet and in which clinical manifestations reappeared after the challenge. Lastly, the case [75] of a 10-day-old child with enteropathy signs and symptoms and eosinophil infiltrate on antral, duodenal, jejunal, and colic biopsy was reported, who, after discontinuation of maternal breast milk feeding and the start of an EHF, no longer presented clinical manifestations and went into histological remission.

Therefore, because of the paucity of data available, the elimination diet in breastfeeding mothers of infants with FPE does not seem to play a relevant role.

## 5. Management of a Suspected Food Allergy in Exclusively Breastfed Infants

**Figure 2:**
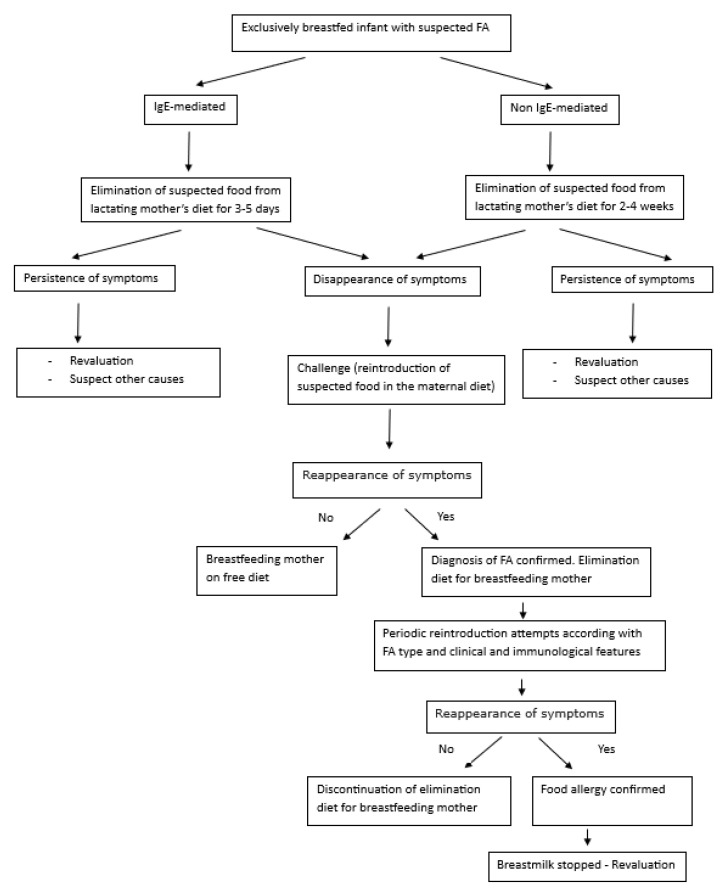
Algorithm for the management of a suspected FA in exclusively breastfed infants.

Food allergy in exclusively breastfed infants is quite rare, and any effort should be made to avoid potential harm associated with the overdiagnosis of this condition. The evidence base to support the use of elimination diets in mothers to treat breastfed infants with FA is much-debated due to a lack of randomized controlled trials and differential secretion of food allergens in human milk, irrespective of how much of the food allergen a lactating parent is consuming [77]. In Figure 2, we propose an algorithm for managing a suspected food allergy in exclusively breastfed infants based on elimination diets and challenges when needed. In general, the timing of reintroducing the culprit food in the lactating mother of exclusively breastfed infants to assess the acquisition of tolerance once the FA diagnosis has been challenge-proven is not well-defined. According to some suggestions, the food elimination diet can last from 3 months for FPIAP [5] to 6 months (i.e., until weaning) for non-IgE-mediated CMPA [78]. Tolerance of CM (or other food triggers) is usually reached at the age of 1 [77]. The other FAs can reasonably be similar in relation to the severity of the signs and symptoms and other clinical and allergological features. In any case, an effort should be made to limit the duration of the elimination diet with periodic allergen reintroduction temptations.

Although some guidelines suggest completely eliminating offending proteins from breastfeeding mothers’ diets, it must be considered that very small amounts ingested by the mother through products with precautionary allergen labels, such as “this product may contain trace amounts of allergen”, are unlikely to be antigenically relevant to the child. Furthermore, particularly restrictive diets are difficult to follow for mothers and may lead to nutritional deficiencies.

## 6. Conclusions

Breast milk is the most important nutritional source for all infants, and it should also be encouraged for those with food allergies. Apart from selected cases, breastfeeding mothers should observe a free and adequate diet according to their nutritional needs. Several food allergens are found in human milk, but their clinical significance and role in immune modulation are still uncertain, although a very small number of cases have been identified as causing allergies in breastfed infants. Dietary restrictions in lactating mothers of infants with suspected FA are usually not necessary. Only if significant allergy signs and symptoms occur in the infant during exclusive breastfeeding should the lactating mother be placed on an elimination diet for the suspected food for a short period (3 days to 4 weeks). If clinical manifestations disappear, a challenge should always be carried out to avoid unnecessary dietary restrictions harmful to the mother. Diagnosis of food allergy through breast milk is only made in the case of a reappearance of symptoms during the challenge. Any effort should be made to shorten the duration of any elimination diet with periodic allergen reintroduction temptations for all of the breastfeeding time according to the FA type and clinical and immunological features.

## Figures and Tables

**Table 1:** Current recommendations provided by international guidelines regarding the elimination of diets in lactating mothers.

Guidelines/Recommendations	Year of Publication	Advice Regarding Breastfed Infants
BSACI guideline for the diagnosis and management of cow’s milk allergy [1]	2014	Breast milk is advisable for most infants with CMA. Therefore, breastfeeding should be encouraged, usually with no dietary dairy restrictions unless the infant shows symptoms while being breastfed. Nonetheless, small amounts of cow’s milk proteins found in breast milk can elicit symptoms in exclusively breastfed infants never receiving cow’s milk.
International consensus guidelines for the diagnosis and management of food-protein-induced enterocolitis syndrome [2]	2017	Dietary elimination of offending triggers must not be recommended in lactating mothers if the infant is thriving and remains asymptomatic. In the case of symptomatic FPIES occurring in an exclusively breastfed infant, the trigger food or foods should be eliminated from the mother’s diet if reactions occur after breastfeeding or if the infant fails to thrive. Do not routinely recommend avoiding products with precautionary allergen labels in patients with FPIES.
Committee on Nutrition of the French Society of Pediatrics [3]	2018	Breast milk is preferable for all infants, including those with CMA. Milk and dairy products should be removed from the mother’s diet if the child is diagnosed with CMA when breastfed.
Diagnosis and management of non-IgE gastrointestinal allergies in breastfed infants—An EAACI Position Paper [4]	2019	Maternal elimination diets for 2–4 weeks with symptom improvement or resolution, followed by reintroduction with symptom deterioration, remains the cornerstone for diagnosis. does not apply in the case of a convincing history of FPIES or severe associated symptoms. The symptoms are present when reintroduction does not occur. International FPIES guidelines do not recommend routine allergen avoidance in breastfeeding mothers unless a child presents with symptoms while breastfeeding. In such cases, avoidance may be necessary. FPIAP: treatment, if required, is based on strict exclusion of the culprit food in the mother’s diet, usually cow’s milk, but other dietary antigens may also need to be eliminated. FPE: When food protein is suspected, a maternal elimination diet should be implemented, followed by reintroduction.
WAO food-protein-induced allergic proctocolitis in infants: literature review and proposal of a management protocol [5]	2020	In breastfeeding infants, it is vital to support the beneficial role of breastfeeding. Yet, cow’s milk proteins should be eliminated from the maternal diet. Clinical bleeding typically resolves within 1 to 2 weeks of eliminating the offending protein from the mother’s diet.
WAO DRACMA guideline group [6]	2023	In the current clinical practice recommendations, a 2–4-week trial of maternal cow’s milk dietary elimination is advised for the following: (a) IgE-mediated cow’s milk allergy, if the infant is symptomatic while breastfeeding alone; (b) Non-IgE-mediated associated symptoms, if the history and examination strongly point at cow’s milk allergy; (c) Infants with moderate to severe eczema/atopic dermatitis not responding to topical steroids and sensitized to cow’s milk protein. There should be a clear plan to reintroduce cow’s milk into the maternal diet at home for 1 week in order to determine a cause–effect relationship between the cow’s milk elimination and the resolution of symptoms. Then, there is a subsequent reoccurrence of infant symptoms upon maternal cow’s milk reintroduction. The evidence base to support avoiding cow’s milk in the mother’s diet so as to treat a breastfed infant with cow’s milk allergy is limited due to a lack of high-quality, adequately powered, randomized controlled trials.
An ESPGHAN position paper on the diagnosis, management, and prevention of cow milk allergy [7]	2024	CMA in exclusively breastfed infants is a rare condition. Dietary restrictions in breastfeeding mothers are usually not needed. Therefore, in exclusively breastfed infants with chronic symptoms of CMA dietary restrictions should only be considered in rare and specific circumstances. Clinical trials do not consistently support the exclusion of CM from the maternal or infant diet to manage common symptoms in infants without demonstrated CMA. Up to 20% of breastfed infants show spontaneous resolution of symptoms, such as rectal bleeding, without changing their mothers’ diets. Breastfeeding with a maternal elimination diet for CM may be considered for 2 to 4 weeks.

## Abbreviations

FA	food allergy
CMPA	cow’s milk protein allergy
CM	cow’s milk
EAACI	European Association of Allergy and Immunology
EHF	extensively hydrolyzed formula
AAF	amino acid formula
FPIAP	food-protein-induced allergic proctocolitis
FPIES	food-protein-induced enterocolitis syndrome
OFC	oral food challenge
FPE	food protein enteropathy
